# Development of Nursing Research in Saudi Arabia: Implications for Policies and Practice

**DOI:** 10.3390/nursrep13030104

**Published:** 2023-09-09

**Authors:** Ahmad Aboshaiqah, Kamila Alammar, Ali Alenezi, Bandar Majrashi, Yousef Alshamlani, Abdullah Alshehari, Naif H. Alanazi

**Affiliations:** 1 Nursing Administration and Education Department, College of Nursing, King Saud University, King Khalid Road, Riyadh 11421, Saudi Arabia; 2 SURGCA, Dar Tala Medical Care, Riyadh 13317, Saudi Arabia; kalammar89@gmail.com; 3 King Fahad Hospital in Madinah, Madinah al-Munawwarah 42351, Saudi Arabia; amalenezi@moh.gov.sa; 4 King Saud Medical City, Riyadh 3617, Saudi Arabia; bmajrashi@moh.gov.sa; 5 Nursing Department, King Saud University Medical City, Riyadh 12372, Saudi Arabia; yalshamlani@ksu.edu.sa; 6 Khamiss Mushayt Hospital, Aseer 62433, Saudi Arabia; alshehari@moh.gov.sa; 7 Medical-Surgical Department, College of Nursing, King Saud University, Riyadh 12372, Saudi Arabia; nalanazz@ksu.edu.sa

**Keywords:** nursing, research, Saudi Arabia

## Abstract

**Background:** Nursing research in Saudi Arabia can be evaluated based on productivity as well as the quality of publications. The scope of scientific inquiry in nursing research expands to include clinical, health system, and outcome-based research, education, and administration. **Aim:** The purpose of this article is to track the development of nursing research in the Kingdom of Saudi Arabia. **Design:** Systematic review. **Methods:** This study used keywords, databases including MEDLINE, CINAHL, and PubMed to search for published articles on nursing in Saudi Arabia. The search resulted in the identification of 681 publications, from which 360 articles met the inclusion criteria and were included in the review. **Results:** The highest percentage of studies (56.7% of articles) focused on nursing clinical practice, and 76.0% of the studies were conducted in a hospital setting, followed by an educational setting. Most of the studies were quantitative and non-funded. More than 50.0% of the studies were first authored by Saudi scholars. **Conclusions:** This study concluded that nursing research in Saudi Arabia is still in its infancy, with notable improvements in the last 5 years. This correlated with an increasing number of nurses holding postgraduate degrees. With the Saudi government’s strong support, the number of scientific research papers published on Saudi nursing has steadily increased over the last year.

## Impact Statement

This review provides a comprehensive analysis of the state of nursing research in Saudi Arabia and recommendations for policy and practice. It indicates the need to promote a nursing research culture and improve nursing publication output in Saudi Arabia.

## Plain Language Summary

Nurses are crucial to the healthcare system and vital in promoting health and providing patient care. Nursing research is essential for advancing nursing knowledge, improving patient outcomes, and informing policy and practice. We reviewed the published nursing research from Saudi Arabia to outline its state by tracking nursing research development. Our findings indicate the recent growth of nursing research in Saudi Arabia. Governmental funding support and an increasing number of nurses holding postgraduate degrees contribute to this growth. However, the field of nursing research in Saudi Arabia is still in its early stages of development. Therefore, the implication for policy and practice was recommended.

## 1. Introduction

The role of a nurse has changed dramatically, since its extension beyond the hospital setting, to reach schools, communities, homecare, and businesses [1], aiming to provide high-quality evidence-based care. Nursing, as a profession, has been legitimized through research [2]. Nursing research is a systematic process that not only validates and refines existing knowledge but also analyzes phenomena of importance to nursing, which encompasses nursing education, administration, healthcare delivery, characteristics of nurses, and their roles [3,4]. 

However, people have not taken nurses’ roles as seriously as other professional health fields [5]. Nevertheless, nursing research plays a significant role in advancing nursing practice and shaping health policy [6]. The main purpose of research in nursing is to ensure quality care based on other factors such as cultural beliefs and other forms of patient care [7]. Change can be driven by the research conducted by nurses and the contributions they make as members of multidisciplinary research teams. Research findings affect and shape the nursing profession, as well as inform and support policy, professional decision making, and nursing actions. It is the foundation of the high-quality evidence base in nursing [8]. 

Nursing research has a rich history, commencing with Nightingale’s pivotal studies in the 1850s that linked nurse education to quality patient care [9]. Her foundational work during the Crimean War paved the way for environmental modifications to patient care settings [10]. Immediately after the Nightingale era, little literature about nursing research can be found [11]. From the early 1900s through 1950, nursing research focused on public health, care plans for specific groups of patients, and the organization and delivery of healthcare [12]. In 1960, nursing research turned its attention toward the patient-centered research, prompting nurse researchers to study not only practice methods but also their impact on patient outcomes [12]. However, it was noticed that nursing research was influenced by sociologists’ and psychologists’ work [12]. A significant shift in the late 20th century saw nursing research evolve from descriptive studies to more explanatory and predictive methodologies, often integrating both qualitative and quantitative methods [11]. Presently, nursing research embraces a multidisciplinary approach, reflecting its growing breadth and complexity [12]. The scope of scientific inquiry in nursing research expands to include clinical, health system, and outcome-based research, education, and administration [6,13,14]. According to the American Association of Colleges of Nursing (2006), clinical research is concerned with the treatment of individuals in a community and conducts research in any clinical setting [6]. Health systems and outcomes research analyzes a variety of factors affecting the quality, quantity, and cost of healthcare services and makes recommendations for how to enhance healthcare delivery [6]. While nursing education research is focused on the faculty and students, it is also concerned with the science of learning and teaching [15]. Finally, nursing administrative research emphasized nurse management practice, nursing ethics, and nursing attributes associated with work satisfaction, employee retention, patient satisfaction, care quality, and cost [16].

Nursing research has advanced more rapidly in high-income countries than in many developing countries, particularly Arab ones [17]. However, over the last four decades, the number of Arab nursing publications has increased, particularly in Jordan, followed by Saudi Arabia and Egypt [18].

In Saudi Arabia, the *Saudi Vision 2030* initiative places a premium on research and development, with the objective of attaining an international level in higher education [19]. As a result, the Nature Index report 2021 stated that Saudi Arabia is the leader in scientific research among Arab countries, as it ranks in the top 50 countries in the world with the highest share of scientific research. However, among the health science research, nursing contributed less research than the other areas in Saudi Arabia, with 1.6% of the total health sciences research publications [19]. King Saud University, King Abdulaziz University, and King Faisal Specialist Hospital and Research Centre all rank among the top 15 most productive Arab institutions in terms of nursing publishing, ranking 5, 11, and 15, respectively [18]. Up to date, the situation of Saudi nursing research has not been explored yet. This review paper will attempt to provide an outline of the state of nursing research through tracking the development of nursing research in Saudi Arabia. We aimed to answer the following question: In nursing researchers in Saudi Arabia, how has the conduct of nursing research influenced the current state and historical development of nursing research, compared to the development in other countries or regions?

## 2. Methods

### 2.1. Study Design 

This literature review was carried out based on the preferred reporting items for systematic reviews and meta-analyses (PRISMA) procedure [20] to track the development of nursing research in Saudi Arabia. 

### 2.2. Ethical Consideration

This study is a review paper based on previously published articles. Thus, this study did not require approval from the Institutional Review Board or Ethical Committee.

### 2.3. Search Methods 

An extensive literature review was conducted using electronic database searches of MEDLINE, CINAHL, the Cochrane Library, and PubMed to retrieve studies which were used to search for published Saudi nursing articles. A preliminary search was conducted to identify relevant keywords to frame an advanced search among the mentioned databases. The identified keywords were: ‘nursing’, ‘Saudi’, and ‘healthcare’. Synonyms from the database glossary (MeSH terms) were then incorporated to ensure all synonym terms (e.g., nurs*) were identified through the database search.

### 2.4. Inclusion and Exclusion Criteria

A critical search was conducted for all published articles related to nursing in Saudi Arabia, with no limitation to a starting year and published through to March 2021. The articles needed to be available in full text, published in English, as per the English language standards for nursing in Saudi Arabia, and conducted in Saudi Arabia. According to the inclusion and exclusion criteria used during the search, dissertations, conference abstracts, symposiums, and editorials were excluded from this review, as well as the grey literature that is not controlled by commercial publishers. 

### 2.5. Screening of Articles

Mendeley was applied to import and manage the search results. After deleting the duplicates, two reviewers independently evaluated the studies by title and abstract and examined the full text of each study. All disagreements were resolved by discussion. 

### 2.6. Data Extraction 

A Microsoft Excel spreadsheet (Microsoft Corporation, Redmond, WA, USA) used to extract and collect the essential data required from each article. The required data were added to the template, which includes funding, specialty, author, design, country, participants, setting, framework, sample, and year of publication. The identified articles were distributed between the researchers to perform a critical evaluation and data-collection process which included the full article, extracting data, and then entering the extracted data into the template.

### 2.7. Validity of the Data

To ensure the validity of the data, we performed a critical review of the extracted data by two independent reviewers, and some discrepancies were identified. We solved this issue through further clarification and discussion, which included re-reviewing the articles, discussion among the researchers, and re-entering the data. Additionally, discrepancies in evaluation were resolved by a third senior reviewer.

### 2.8. Data Synthesis and Analysis

The data were analyzed by utilizing the simple descriptive statistics to quantify and describe the basic characteristics of the selected studies. 

## 3. Results

### 3.1. Search Result 

**Figure 1:**
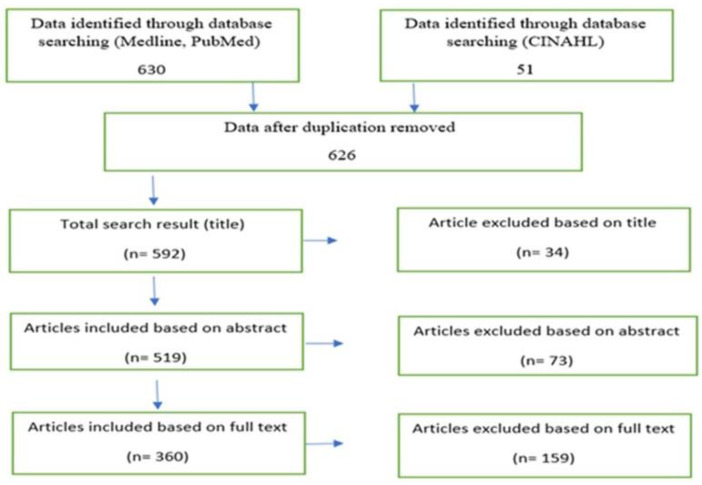
PRISMA flow chart summarizing the search results developing nursing research in Saudi.

A total of 681 articles were identified during the initial search. These identified articles were analyzed based on the PRISMA flow diagram (Figure 1). This analysis resulted in the removal of 55 articles due to duplication, 34 articles due to the title not being relevant, 73 articles were rejected for abstract review, and 159 articles were rejected for full-text review (Figure 1). As a result, the total number of retrieved articles after the analysis was 360. The excluded articles do not conform to: the first author being a Saudi national; the first and/or coauthor being a Saudi nurse, and the topic being purely nursing. 

### 3.2. Nursing Research, According to the Year of Publication

As can be seen in Supporting Information Table S1, a review of the literature revealed that the first article was published in 1985, followed by two more in 1988, all of which discussed the history of nursing education in Saudi Arabia. However, none of these papers was based on research, and they were all written by non-Saudis. The first research-based publication was produced in 1991, by two western nurses who worked at King Faisal Specialist Hospital and Research Centre. It discussed the attitudes and perceptions of secondary and university Saudi students toward the healthcare system and nursing professions in Saudi Arabia. In the 1990s, research progressed slowly, with only nine articles published between 1991 and 2000. In the period between 2001 and 2010, only 24 papers were published, which is considered to be a remarkably slow rate of progress. In the period from 2011 to 2016, 86 articles were published, which is considered the first milestone of the growing body of research in Saudi Arabia. The last five years (2017–2021) might be characterized as a considerable increase in research activities, with 238 articles.

### 3.3. Nursing Research According to the Setting of Data Collection

Most of the published articles 75.6% (*n* = 272) were undertaken in the hospital setting, followed by 15.2% (*n* = 55) completed in an educational setting, and 3.4% (*n* = 12) were undertaken in community and primary care settings. The settings for the remaining articles were not identified.

### 3.4. Nursing Research According to Study Design

Almost seventy-one percent of the articles used a quantitative method, while 20.5% used a qualitative approach. Mixed approaches were used in approximately 3.6% of the articles. Systematic reviews constituted approximately 5.3% of the articles. A descriptive design was used in most quantitative studies, while the highest percentage of the qualitative studies utilized a phenomenological approach. 

### 3.5. Nursing Research According to the Participants

Nurses were the most selected participants by more than 63.0% as the only subjects of the study, followed by students by 15.8% (*n* = 57), healthcare providers by 5.0% (*n* = 18), and notably only 7.0% of the participants in the studies were patients. The details can be found in Supplementary Table S2.

### 3.6. Nursing Research According to Sample Types

Convenience sampling (*n* = 192) was the most common method used by 53.3%, followed by no sampling by 16.7% (*n* = 60), purposive sampling by 9.7% (*n* = 35), and random sampling by 11.0% (*n* = 40), while 6.6% (*n* = 24) of the studies did not explain the method of sampling. 

### 3.7. Nursing Research According to the Utilization of a Framework

The literature review revealed that most nursing researchers did not use any framework (83.3%; *n* = 300), a conceptual framework was used by 9.7% (*n* = 35), and a theoretical framework was used by 7.0% (*n* = 25).

### 3.8. Nursing Research with the Funding Source

Approximately 34.2% (*n* = 123) did not disclose information about funding, 42.5% (*n* = 153) of the published research was not funded, 14.7% (*n* = 53) was funded by an educational source, 7.2% (*n* = 26) was funded by hospitals, and 1.4% (*n* = 5) was funded by other sources such as charities. 

### 3.9. Nursing Research According to the Research Topic Selected

Most published articles were focused on nursing clinical practice at 56.7% (*n* = 204), followed by nursing management and administration issues in 20.0% (*n* = 72). Studies about nursing education were 17.5% (*n* = 63) of the total. Studies about nursing quality and history were 2.5% and 2.2%, respectively. Less than 1.0% (*n* = 3) of the publications were concerned about research methodologies. The details can be found in Supplementary Table S3.

### 3.10. Nursing Research According to Authorship

Most nursing research was first authored by Saudi scholars (53.6%; *n* = 193), 23.3% (*n* = 84) was authored by non-Saudi scholars, and 17.0% (*n* = 61) was co-authored by a Saudi nurse; approximately 6.1% was authored by non-nurses (*n* = 22).

### 3.11. Nursing Research According to Country of Data Collection

Most of the studies were conducted in Saudi Arabia 82.0% (*n* = 296), followed by 2.0% (*n* = 8) of articles from Jordan, as well as the United States. Furthermore, 2.5% (*n* = 9) of articles were carried out in multiple Asian countries such as the Philippines, Malaysia, and China. Only two studies were conducted in the UK and Australia. Thirteen studies from the selected articles were conducted in multiple countries as the setting for each study (3.6%). The lowest percentage, 0.3% (*n* = 1 each), of studies was conducted with a sample from the United Arab of Emirates and Canada. Systematic reviews made up about 5.0% of the publications.

## 4. Discussion

The literature review revealed that nursing research in Saudi Arabia is still in its infancy. The first published paper was in 1985, but postgraduate nursing education started with the establishment of the first master’s degree in 1987 at King Saud University. However, the first scientific research began in 1991. According to Ul Haq et al. (2020), nursing research productivity is considered the lowest among health sciences publications from Saudi Arabia [19].

Contrasting this with international developments, nursing research in countries like the United States and Europe began as early as the 1950s [11,21,22]. Interestingly, Jordan emerged as an early leader in Arab nursing research with its first paper published in 1951 [23]. Our study also highlights the predominant focus on hospital settings in Saudi Arabia’s nursing research, with limited emphasis on community and primary care settings. This trend appears misaligned with Saudi Arabia’s 2030 vision, which stresses enhancing primary care and community services [24]. It underscores a pressing need for more research in community healthcare to foster public health improvements and resonate with the Health Sector Transformation Program’s aspirations. According to Elmorshedy et al. (2020), in Saudi Arabia, most published studies focused on nursing students and graduates [25]. However, our findings demonstrate that most of the published studies in Saudi Arabia were focused on nurses in clinical settings. This finding aligns with the results of Deeb AM and Aljuaid MH (2020), which showed that most of the published critical care nursing articles in Saudi Arabia focused on clinical practice settings [26]. 

Researchers in health promotion argue that nurses have yet to exhibit a clear and visible political role in the implementation of health promotion initiatives [27]. Nurses, on the other hand, might be regarded as general health promoters, as their efforts are based on sound knowledge and providing information to patients. Although nursing is an excellent profession for promoting health, various limitations linked to organizational culture have a significant impact on delivery. As a result, further research is needed to figure out how to help nurses adopt health promotion in their many responsibilities in healthcare settings.

Concurrent with Borbasi et al. (2002), our study showed that the majority of nursing research in Saudi Arabia focuses toward descriptive research, while interventional studies are limited [28]. Given Saudi Arabia’s emphasis on healthcare advancement, there is potential for impactful nursing intervention research [29]. Such studies, especially if contextually crafted, can significantly influence healthcare quality. The result of this study shows a low percentage of the use of nursing theory compared with the study reported by Bond et al. (2011), which showed that more than 50% of their studies used nursing theory [30]. In addition, 61.6% of the study participants were nurses, while only 6.6% of the participants were patients. This study shows a similar result to the Australian study reported by Borbasi et al. (2002), which indicates that most of the data were collected from nurses, followed by patients, and that they were not investigating major health issues in Australia [28]. For that reason, the integration and cooperation of the National Center for Public Agencies Performance Measurement, which measures the performance of government agencies, performance indicators of public agencies, and the satisfaction of beneficiaries with government services, with researchers from universities or hospitals will be beneficial for the quality of the healthcare system.

Globally, several factors enhance the growth of nursing research. In the US, the factor that might be contributing to nursing research was the establishment of the National Center for Nursing Research (NCNR) in the United States Public Health Service (USPHS) in 1986, which was the most significant. The American Nurses Association’s vigorous political campaign culminated in the creation of this center (ANA) [31]. Yet, federal funds supporting research in the 1950s were concentrated on the diagnosis and cure of disease for medical studies, before the establishment of the NCNR, which became the National Institute of Nursing Research (NINR) in 1993 with a budget of $16.2 million [32] in 1986 and $156.8 million in 2021 [33].

In contrast, approximately 42% (*n* = 154) of the published nursing research was not funded in Saudi Arabia. Only 23% of published nursing research was funded; 15.8% (*n* = 57) funded by education, 4.4% (*n* = 16) funded by hospitals, and less than 1% (*n* = 3) funded by charity.

Consequently, legislative bodies, regulatory agencies, universities, healthcare facilities, and nursing organizations must collaborate to develop a policy that would improve the culture of nursing research and offer funding to encourage nurses to participate in nursing research in order to develop the profession. Thus, prioritizing the area of research that needs attention and formulating a plan aligned with *Saudi Vision 2030*, the Health Sector Transformation Program will direct nursing researchers in Saudi Arabia. 

Finally, this study has some limitations. It did not attempt to conduct a comprehensive analysis of the published articles. Additionally, it includes articles that were published in indexed journals, which may have missed an additional publication.

### Implications for Nursing Practice and Policies

To meet the need of increasing nursing research productivity, nurses in Saudi Arabia should be actively involved in healthcare research as part of professional practice. This study shows a lack of theoretical guidance. This issue might change over time, taking into consideration the increasing number of nursing PhD holders in Saudi Arabia and the increasing number of research projects in general.

Healthcare improvement is a national goal in Saudi Arabia, supported by the transformational vision and long experience with crowd medicine during pilgrimages; added to that is the experience gained during the last pandemic, COVID-19. For that reason, nursing research is vital for enhancing the health field, specifically interventional research, as it is a contributing factor that significantly influences the progress of the knowledge that underpins high-quality healthcare.

This study shows what was the focus of nursing research in Saudi until now. This may have an impact on what researchers and postgraduate students write about. In addition, it is beneficial to increase the funds from different organizations for a specific type of research that is needed and for researchers in general. 

## 5. Impact Paragraph

Nurses should actively participate in healthcare research as part of professional and academic practices. This review provides a comprehensive analysis of the state of nursing research in Saudi Arabia and recommendations for policy and practice. Although nursing research has grown in recent years, it is still immature compared to developed countries. Greater emphasis should be placed on developing more nursing research, especially in community health. This review indicates the need to promote a nursing research culture and improve nursing publication output in Saudi Arabia.

## 6. Conclusions

There has been a gradual improvement in nursing research in Saudi Arabia since the first publication in 1985. The milestone of that growth was between 2011 and 2016, with 86 articles. However, nursing research in Saudi Arabia is still in its infancy. This study shows a low percentage of the use of nursing theory or a framework, at only 6.9% of the studies. There is a need for researchers to integrate recognized theoretical models, which could enhance the depth and validity of their findings. Most of the published research in Saudi Arabia was focused on registered nurses. Efforts need to be put towards conducting research studies in more diverse areas such as the community field. Nursing leaders need to develop policy that promotes a culture of nursing research and improves nursing publication output in Saudi Arabia. To advance nursing research in line with the Saudi Vision 2030, there is an imperative need for collaboration among legislative bodies, universities, and healthcare institutions. Together, they should establish policies, prioritize areas of research, and secure funding to promote and support nurse researchers.

## Data Availability

Not applicable.

## Supplementary Materials

The following supporting information can be downloaded at: https://www.mdpi.com/article/10.3390/nursrep13030104/s1, Table S1: Number and percentage of research according to year of publication, Table S2: Number and percentage of published research according to the participants, Table S3: Number and percentage of published research according to research topic, File S1: PRISMA Checklist.

## Public Involvement Statement

No Patient or Public involvement.

## Guidelines and Standards Statement

This literature review was carried out based on the preferred reporting items for systematic reviews and meta-analyses (PRISMA) procedure [20].
