# Supplementary material for: Development of Nursing Research in Saudi Arabia: Implications for Policies and Practice
Source: Nurs Rep. 2023 Sep 9;13(3):1216–24. doi: 10.3390/nursrep13030104 (PMC10536154; doi:10.3390/nursrep13030104)
Supplement: Supplementary file 1 [file nursrep-13-00104-s001.zip › supplementary materia.pdf]

Table 1: Number and percentage of research according to year of publication

Year	Frequency	Percent
**1985**	1	.3
**1988**	2	.6
**1991**	1	.3
**1992**	1	.3
**1996**	1	.3
**1998**	2	.6
**1999**	1	.3
**2000**	3	.8
**2001**	3	.8
**2002**	1	.3
**2005**	1	.3
**2006**	3	.8
**2007**	3	.8
**2008**	4	1.1
**2009**	4	1.1
**2010**	5	1.4
**2011**	4	1.1
**2012**	17	4.7
**2013**	8	2.2
**2014**	14	3.9
**2015**	15	4.2

**2016**	28	7.8
**2017**	33	9.2
**2018**	79	21.9
**2019**	62	17.2
**2020**	51	14.2
**2021**	13	3.6
**Total**	360	100.0

Table 2: Number and percentage of published research according to the participants

Participants	Number	Percentage
**Nurses**	227	63
**Students**	57	15.8
**Nurses and other participants**	9	2.5
**Healthcare providers**	18	5
**Patients**	25	7
**No participants**	24	6.7
**Total**	360	100

Table 3: Number and percentage of published research according to research topic

Reseach topic	Number	Percentage
**Nursing Clinical Practice**	204	56.7
**Nursing Administration**	72	20
**Nursing Education**	63	17.5
**Nursing History**	8	2.2
**Nursing Quality**	9	2.5
**Research and methodology**	3	0.83
**Not Nursing**	1	0.27
**Total**	360	100

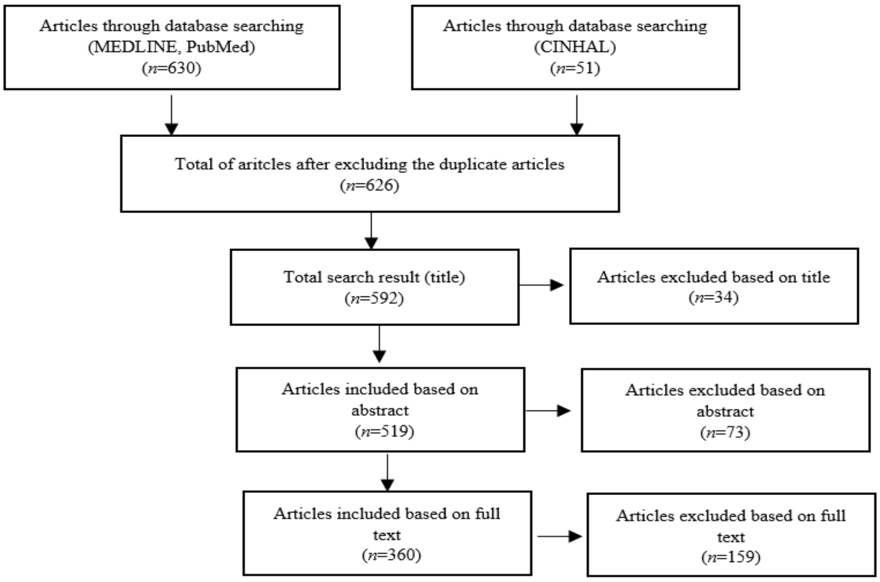

Figure. 1 PRISMA Flow Diagram
